# Differences in topographical location of sacroiliac joint MRI lesions in patients with early axial spondyloarthritis and mechanical back pain

**DOI:** 10.1186/s13075-022-02760-7

**Published:** 2022-03-24

**Authors:** Rosa Marie Kiil, Clara E. Mistegaard, Anne Gitte Loft, Anna Zejden, Oliver Hendricks, Anne Grethe Jurik

**Affiliations:** 1 Department of Radiology, Aarhus University Hospital, Palle Juul-Jensens Boulevard 35, C105, 8200 Aarhus N, Denmark; 2 Department of Clinical Medicine, Aarhus University, Palle Juul-Jensens Boulevard 103, 8200 Aarhus N, Denmark; 3 Institute of Regional Health Research, University of Southern Denmark, Winsloewparken 19, 5000 Odense C, Denmark; 4 Department of Rheumatology, Aarhus University Hospital, Palle Juul-Jensens Boulevard 59, 8200 Aarhus N, Denmark; 5 Danish Hospital for Rheumatic Diseases, Engelshøjgade 9A, 6400 Sønderborg, Denmark

**Keywords:** Spondyloarthritis, Back pain, Imaging, Magnetic resonance imaging (MRI)

## Abstract

**Background:**

Early diagnostics of axial spondyloarthritis (axSpA) remains a challenge. Traditional imaging one-plane sacroiliac joint (SIJ) MRI assessment is used. By introducing a two-plane assessment system, the objective was to analyse the differences in SIJ MRI changes in early axSpA compared with changes in patients with mechanical back pain (MBP) by exploring the differences in volume and location.

**Methods:**

MRIs in the early diagnostic state of 25 axSpA patients (mean age 31.3 years) and 59 MBP patients (mean age 32.3 years) were included. The MRIs were assessed by two readers regarding the distribution of bone marrow edema (BME) in 14 joint portions and structural changes in six joint portions in addition to SIJ anatomical variations and lumbar spine disc degeneration.

**Results:**

AxSpA patients had a significantly higher overall BME sumscore (volume) of 25.1 compared to MBP patients 6.8, *p* < 0.005. The MBP group had the highest prevalence (66%) and sumscore (5.7) in the middle anterior sacrum. The axSpA group had significantly higher prevalence and sumscores in all joint portions except the three cartilaginous anterior sacral joint portions, including the ligamentous compartments (prevalence 40–60% compared to 8–15%, *p* both < 0.005). The axSpA group had also a significantly higher prevalence of erosions and fatty marrow disposition, but there were no differences in the prevalence of anatomical variations except the bipartite iliac bony plate.

**Conclusions:**

AxSpA patients demonstrated a widespread distribution of both inflammatory and structural changes, including high BME occurrence in the ligamentous joint portions whereas patients with MBP had the highest occurrence of BME in the middle anterior sacrum. These findings may help differentiate axSpA patients from other back pain conditions in the early diagnostic phase.

## Background

Sacroiliitis is a hallmark in axial spondyloarthritis (axSpA) where the active and often reversible magnetic resonance imaging (MRI) finding, subchondral bone marrow edema (BME) around the sacroiliac joints (SIJ), is interpreted as active sacroiliitis. The majority of axSpA patients have BME on SIJ MRI [1, 2], which plays a major role in the widely used 2009 Assessment of SpondyloArthritis International Society (ASAS) classification criteria for axSpA [3, 4]. Unfortunately, BME is not exclusively seen in axSpA; previous studies have demonstrated that up to 25% of patients with low back pain, and even healthy individuals, have non-specific BME on SIJ MRI [5–8] with even higher prevalence rates in pregnant and post-partum women with or without low back or SIJ pain [1, 2, 6, 9–11].

Despite the apparent low specificity of BME, comparative studies regarding the volume and topographic distribution of BME and other MRI lesions in axSpA patients versus various groups of individuals with and without back pain are sparse. The comparative approach of these diagnostic entities may contribute to the differentiation of this clinically highly relevant and difficult challenge. It has been reported that the location of BME in healthy subjects, patients with mechanical back pain (MBP) and postpartum females is most frequent in the lower ilium and anterior upper sacrum with minimal accompanying structural changes like erosions and fatty lesions [6, 12]. However, another study did not find any differences in the distribution [2]. The distribution of BME in axSpA patients has been reported being located more widespread in both the ilium and the sacrum as well as being more voluminous and accompanied by structural changes [6, 8, 12, 13].

The BME occurring in non-SpA individuals may be due to degenerative changes [14, 15], mechanical load [7] and atypical SIJ morphologies including lumbosacral transitional anomaly [16–19], which however also can influence the MRI findings in axSpA patients.

Traditional, one-plane SIJ MRI assessment is used in both the diagnostic [4] and monitoring phase [20] of axSpA, which can limit the precise location of lesions and distinction between the cartilaginous and ligamentous joint compartments.

The aim of this study was to analyse the differences in SIJ MRI changes in early axSpA patients compared with changes in patients with MBP by exploring the differences in volume and distribution pattern of MRI SIJ findings by using a detailed two-plane quantitative scoring system. Furthermore, to analyse whether certain SIJ MRI changes were independently associated with the following conditions: axSpA, anatomical variations, disc degeneration, parity, age, gender and BMI.

## Methods

### Study sample

The study sample is described in detail elsewhere [21]. In brief, 84 patients were included. They were referred from a cohort of 1020 patients with low back pain for 2–12 months not responding adequately to conservative treatment [22]. They were characterized by either positive MRI according to the 2009 ASAS MRI criteria, fulfilling at least the minimum requirement [4], or a positive HLA-B27 and ≥ 1 clinical spondyloarthritis (SpA) feature according to the ASAS classification criteria [3]. These inclusion criteria were chosen to ensure that all potential axSpA patients in the basic cohort were included in the study sample. The diagnostic progress involved a retrospective evaluation by multidisciplinary team conferences including both expert rheumatologists and radiologists regarding an axSpA diagnosis after a mean follow-up period of 3.5 years. One of rheumatologists (AGL) had personally assessed 50 (60%) of the 84 included patients clinically at baseline. At the MDT, baseline and mean 3.5-year follow-up MRI of the SIJs and spine as well as clinical and biochemical data were available in all patients. After presentation and discussion on each case, the MDT conference resulted in a definite consensus diagnosis of whether the patient had axSpA or not; 25 (30%) patients were diagnosed with axSpA, whereas the remaining 59 (70%) constitute the group of patients with MBP (Table 1).

**Table 1:** Demographic, biochemical and clinical data. *N* (%) if not otherwise stated

	axSpA (*n* = 25)	MBP (*n* = 59)
Male gender	15 (60%)*	19 (32%)
Age (years), mean (SD)	31.6 (5.7)	32.3 (5.8)
HLA-B27 positivity	18 (72%)*	11 (19%)
BMI (kg/m^2^), mean (SD)	26.4 (4.2)	26.5 (4.4)^a^
Childbirths (number), mean (SD) if female (*n* = 50)	1.6 (1.1)	1.7 (1.2)
Time since last childbirth (years), mean (SD) (*n* = 40)	5.3 (4.2)	4.0 (4.2)
Fulfilment of the 2009 ASAS criteria [3]	24 (96%)*	36 (61%)
Sacroiliitis according to the 2009 ASAS criteria [4]	24 (96%)	49 (83%)
Inflammatory back pain (ASAS definition [3])	20 (80%)*	30 (51%)
Buttock pain	23 (92%)*	41 (69%)

*ASAS* Assessment of SpondyloArthritis International Society, *axSpA* axial spondyloarthritis, *BMI* body mass index, *HLA-B27* human leucocyte antigen subtype B27, *N* number, *SD* standard deviation

*p < 0.05 for test for between-group differences

^a^*n* = 57

### MRI procedure

The same MRI scanner and protocols were used at baseline and follow-up. The scan protocols have been published previously [23]. MRI of the spine and SIJs was performed on a 1.5-Tesla MR scanner (Phillips Achiva). Identical sequences were obtained at baseline and follow-up: SIJ—semi-coronal T1-weighted and T1-fat saturated and semi-axial T2-weighted short tau inversion recovery (STIR) sequences; spine—sagittal T1 and STIR of the entire spine with supplementary 3D T2 Vista sequence of the lumbar spine and axial T2-weighted slices at the three lowest intervertebral spaces. Only the 84 baseline scans were assessed in this study. Two radiologists (one senior consultant specialized in musculoskeletal imaging (AGJ) and one junior resident radiologist (RMK) with 4 years of training in musculoskeletal imaging focusing on SIJ diagnostics) independently performed the granular evaluations of all MR examinations of the SIJ and the lumbar spine blinded to all clinical information. In addition, global assessments of the SIJ MRIs were made independently by two experienced senior musculoskeletal radiologists (AGJ and AZ) blinded to all clinical information regarding the presence of axSpA changes on − 5 (definitely not) to + 5 (definitely yes) confidence scale of the axSpA probability.

### MRI reading

The following SIJ MRI findings were assessed according to the 2019 ASAS lesion definitions [24]: subchondral BME (including depth > 1 cm and intensity), fatty marrow deposition (FMD) (including depth > 1 cm), sclerosis and erosions, along with anatomical SIJ variations (including the presence of BME or FMD/sclerosis in relation to the variations). In the lumbar spine, disc degeneration was evaluated on a 0–3 scale: 0, no signs of disc degeneration or herniation; 1, loss of water content and/or disc height; 2, disc protrusion; and 3, disc extrusion, as defined by Fardon [25]. Besides, the presence of BME and/or FMD was registered divided into vertebral corner and endplate changes, respectively.

The SIJ analyses were based on the simultaneous evaluation of the semi-axial and semi-coronal slices. The iliac and sacral joint facets were divided into three portions based on the semi-axial slices: upper, middle and lower joint portion, regarding the location of BME, FMD, sclerosis and erosion. Furthermore, the location of BME was assessed in relation to the anterior and posterior half of the joint facets.

The joint divisions were determined on semi-axial slices assisted by scout lines on concomitant semi-coronal T1 slices to determine anatomical location. The upper- and lowermost semi-axial slices were determined by the first and last slice with clearly visible SIJ cartilaginous compartment, respectively. Furthermore, a tiny bit of subchondral trabecular bone should be visible at both the sacral and iliac joint facet and extend for ≥ 1 cm on ≥ 1 side. The number of semi-axial slices covering the cartilaginous compartment was divided into three, defining the upper, middle and lower joint portions. In case of an unequal number of slices when dividing by three, one extra slice was added to the lower and eventual middle portions.

For BME, the occurrence and the number of slices per region with changes were registered, thereby obtaining an absolute sumscore. Due to the differences in joint size, e.g. due to gender [26], relative sumscores were used, calculated by dividing the absolute score by the number of slices in the joint portion and for convenience, multiplied by 10.

The presence of FMD, sclerosis and erosions was scored dichotomously as present or absent in each of the region(s). The occurrence of depth > 1 cm of BME and FMD and the intensity of BME (same or almost the same signal intensity as the spinal fluid) were added in each region.

The extent and location of BME were evaluated on semi-axial STIR supported by the semi-coronal T1, whereas the location of FMD, sclerosis and erosions was primarily evaluated on coronal T1 and T1-fat saturated sequences assisted by scout lines on concomitant semi-axial STIR.

BME lesions were registered in both the cartilaginous and the ligamentous compartment, whereas FMD, sclerosis and erosions were only registered in the cartilaginous compartment.

The following atypical SIJ morphologies were reported: accessory SIJ, iliosacral complex, bipartite iliac bony plate and dysmorphic cartilaginous facets. The definitions have been described in detail in a recent paper [19]. Furthermore, the presence of lumbosacral transitional anomaly/anomalies, defined as a vertebra with transverse process (es) articulating with the superior border of the sacrum being either a lumbalization of S1 or a sacralization of L5 [27], was also noted. In addition, BME and structural changes (FMD and/or sclerosis) in relation to the variations were assessed.

For the most prevalent lesions, BME and FMD, the results of the scorings were based on concordant reads, whereas the results regarding the remaining lesions were based on consensus between the readers. BME sumscores were reported as the mean of scores if the readers agreed on the scorings of > 0 in the region. The mean axSpA MRI confidence score was reported.

### Statistical analyses

The interreader reliability on continuous MRI variables was tested by intraclass correlation (ICC) based on a two-way, random effects, single-measure model with the absolute agreements presented, whereas the interreader reliability on binary MRI variables was tested using the kappa test [28]

Demographic and clinical continuous variables were presented as means with standard deviation (SD). Continuous MRI data was presented as mean. The remaining variables were binary and presented as proportions.

The differences between the group prevalence rates were tested with the two-way proportion test for binary variables, whereas the Mann-Whitney rank sum test was used in continuous variables.

Univariate logistic, and when appropriate, linear regressions were performed to investigate whether scoring > 0 uni- or bilaterally for BME, FMD, sclerosis and erosions along with BME scoring > 0 in the ligamentous compartments and the presence of BME and FMD depth were independently associated with an axSpA diagnosis, anatomical variations, lumbar disc extrusion, gender, number of childbirths, age and BMI and were reported as odds ratios (OR) or regression coefficients.

## Results

### Description of the study sample and interreader agreement

Table 1 presents the demographics, clinical, paraclinical and biochemical data on the 84 patients included in the study divided into axSpA patients (*n* = 25) and patients with MBP (*n* = 59). These data, along with further clinical and biochemical data, have been presented in detail in a recent publication [21].

For BME sumscores, disc degenerative changes and the global axSpA MRI confidence score, there were very good agreements with ICC ≥ 0.91 between the two readers whereas ICCs. The kappa values for the presence of BME depth, FMD, FMD depth and atypical morphologies were ≥ 0.81, and thereby almost perfect whereas the kappa values for sclerosis and erosion were ≥ 0.39 and ≥ 0.45, fair and moderate agreements, respectively. The kappa values for BME, FMD and sclerosis in relation to anatomical variations were ≥ 0.39, ≥ 0.23 and ≥ 0.66, respectively.

### Global axSpA MRI confidence score

Table 2 provides the results of the mean confidence scores for the likelihood of axSpA based on the MRI appearance. Eight patients (32%) with the final diagnosis of axSpA had a mean score between − 0.5 and − 4.5, whereas seven (12%) patients with MBP scored between 0.5 and 5.0.

**Table 2:** The mean axial spondyloarthritis (axSpA) confidence scores for the likelihood of axSpA ranging between − 5.0 and 5.0 in MBP and axSpA patients based on MRI assessment only. *N* (%) if not otherwise stated

	axSpA (*n* = 25)	MBP (*n* = 59)
− 5.0 to − 3.0	4 (16%)	36 (61%)
− 2.5 to − 0.5	4 (16%)	12 (20%)
0	2 (8%)	4 (7%)
0.5 to 2.5	2 (8%)	2 (3%)
3.0 to 5.0	13 (52%)	5 (8%)

*axSpA* axial spondyloarthritis, *MBP* mechanical back pain, *N* number

### BME, FMD, sclerosis and erosions

The overall occurrence of BME (> 0 in sumscore in at least one location) was high in both groups being 100% in the axSpA group and 95% in the MBP group. The axSpA group had a higher total sumscore of 25.1 compared to 6.8 (*p* < 0.005) in the MBP group.

**Fig. 1:**
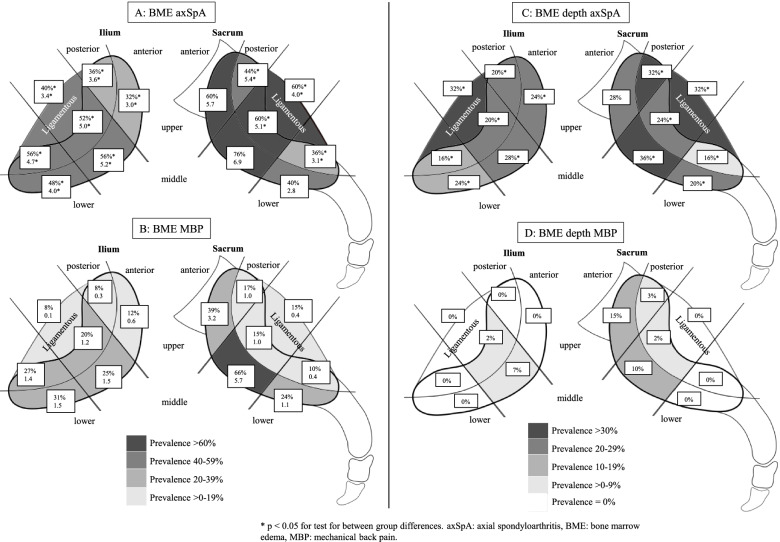
**A**, **B** Prevalence and sumscores of BME in the 12 cartilaginous and two ligamentous joint portions in axSpA (**A**) and MBP (**B**) patients divided in the iliac (left) and sacral (right) sides. **C**, **D** BME depth prevalence in the same locations and patient groups as in **A** and **B**. Boxes in **A** and **B** represent the prevalence and relative sumscore, whereas boxes in **C** and **D** represent the BME depth prevalence in each location

Figure 1A, B presents the prevalence and sumscores of BME in the 14 different locations divided into axSpA (A) and MBP (B) patients. The MBP group had the highest prevalence (66%) and sumscore (5.7) in the middle anterior sacrum, which was also true for the axSpA group. However, the BME distribution in the axSpA group was widespread and equally distributed in the anterior and posterior areas (Fig. 1A). Compared to the MBP group, the axSpA group had significantly higher prevalence and sumscores in all locations, except in three anterior sacral locations.

BME in the ligamentous joint compartments was significantly more frequent in the axSpA group where a BME sumscore > 0 occurred in 60%/40% (sacral/iliac) compared to 15%/8% (sacral/iliac, *p* both < 0.005) in MBP patients.

At least one BME depth score occurred in 60% of axSpA patients. This was significantly more frequent than in the MBP group being 20% (*p* < 0.005). Figure 1C, D presents the BME depth prevalence in the 14 locations divided in axSpA (C) and MBP (D) patients. The prevalence of BME depth was significantly higher in the axSpA group except at the upper anterior sacrum.

Five patients had a BME intensity score, all occurring in axSpA patients.

**Fig. 2:**
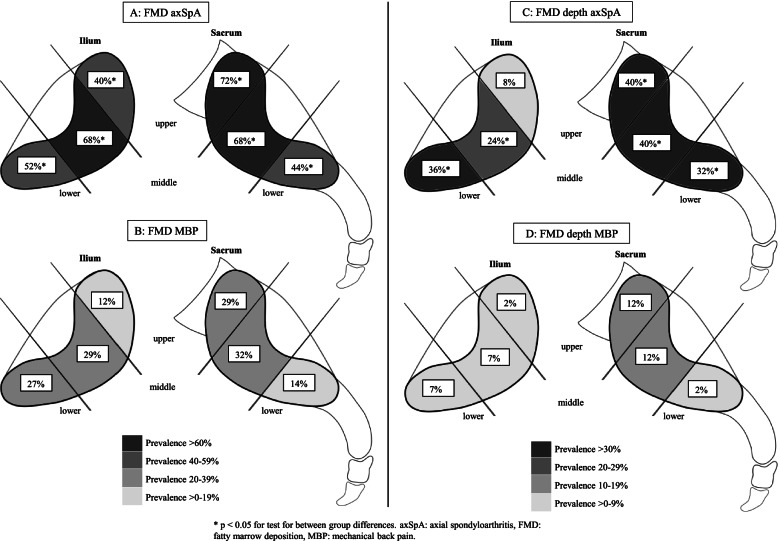
**A**, **B** Prevalence of FMD in the six cartilaginous joint portions in axSpA (**A**) and MBP (**B**) patients divided in iliac the (left) and sacral (right) sides. **C**, **D** Prevalence of FMD depth in the same locations and patient groups as in **A** and **B**. Boxes represent the prevalence in each location

The occurrence of a least one FMD lesion was more frequent in axSpA patients compared to the MBP group, occurring in 76% and 47% (*p* = 0.016), respectively. Figure 2A, B represents the prevalence of FMD in the six cartilaginous locations. AxSpA patients had widespread FMD with significantly higher prevalence in all joint portions compared to MBP patients. MBP patients had the highest FMD prevalence in the middle and upper sacral and middle and lower iliac areas.

The presence of at least one FMD depth score was higher in the axSpA group (52%) compared to the MBP group (19%, *p* < 0.005). Figure 2C, D represents the prevalence of FMD depth in the same locations as Fig. 2A, B. The axSpA group had significantly higher FMD depth prevalence in all locations, except at the upper ilium.

**Fig. 3:**
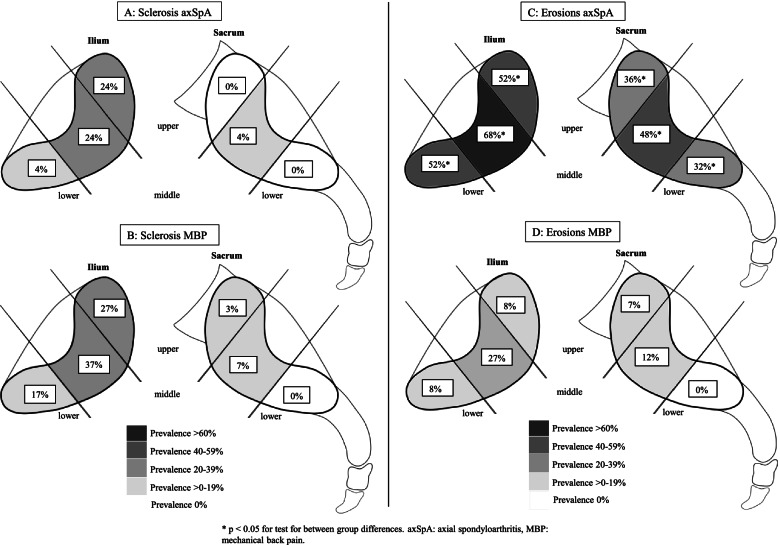
**A**, **B** Prevalence of sclerosis in the six cartilaginous joint portions in axSpA (**A**) and MBP (**B**) patients divided in the iliac (left) and sacral (right) sides. **C**, **D** Prevalence of erosions in the same locations and patient groups as in **A** and **B**. Boxes represent the prevalence in each location

The prevalence of sclerosis and erosions in the six cartilaginous locations are presented in Fig. 3A–D. Subchondral sclerosis was rather similar in prevalence and distribution in axSpA (Fig. 3A) and MBP patients (Fig. 3B) with an occurrence of sclerosis in at least one location of 40% and 42%, respectively. The occurrence of erosions in at least one location was more frequent in axSpA patients compared to MBP patients being present in 80% and 29% (*p* < 0.005), respectively. The axSpA group had a significantly higher occurrence of erosions in all joint portions (Fig. 3C, D) and demonstrated a widespread distribution with the highest prevalence in the three iliac joint portions, whereas the MBP group had the highest prevalence (27%) in the middle iliac joint portion.

### Atypical SIJ morphologies and lumbar spine changes

Table 3 presents the prevalence of four atypical SIJ morphologies, transitional vertebra and disc degenerative changes. The bipartite iliac bony plate was significantly more prevalent in the MBP group, whereas there were no significant between-group differences regarding the remaining variations. Vertebral corner changes (BME and/or FMD) occurred significantly more frequently in axSpA, but there were no between-group differences regarding disc degenerative changes.

**Table 3:** Prevalence of four atypical SIJ morphologies, transitional vertebra and disc degenerative changes. Furthermore, BME and/or structural changes in relation to these. *N* (%) if not otherwise stated

	axSpA (*n* = 25)	MBP (*n* = 59)
Any atypical SIJ morphology	20 (80%)	53 (90%)
Accessory SIJ	9 (36%)	27 (46%)
With BME^a^	6 (67%)	7 (26%)
With structural changes^b^	4 (44%)	9 (33%)
Iliosacral complex	15 (60%)	35 (59%)
With BME^a^	10 (67%)	5 (14%)
With structural changes^b^	5 (33%)	1 (3%)
Dysmorphic cartilaginous facets	10 (40%)	22 (37%)
With BME^a^	10 (100%)	18 (82%)
With structural changes^b^	9 (90%)	9 (41%)
Bipartite iliac bony plate	1 (4%)*	15 (25%)
With BME^a^	0	1 (7%)
With structural changes^b^	0	0
Transitional vertebra	2 (8%)	3 (5%)
With BME^a^	2 (100%)	1 (33%)
With structural changes^b^	2 (100%)	3 (100%)
Disc degeneration (0–3)	1.6	1.7
Disc extrusion (disc degeneration = 3)	6 (24%)	16 (27%)
Vertebral corner changes (BME/FMD)	3 (12%)*	1 (2%)
Vertebral end plate changes (BME/FMD)	6 (24%)	20 (34%)

*axSpA* axial spondylarthritis, *BME* bone marrow edema, *MBP* mechanical back pain, *N* number, *SIJ* sacroiliac joint

*p < 0.05 for the test for between-group differences

^a^ Percent of atypical morphologies accompanied by BME

^b^ Percent of atypical morphologies with structural changes in form of FMD and/or sclerosis in relation to an atypical morphology

### Associations between MRI patterns and axSpA diagnosis, anatomical variations, gender, childbirths, age and BMI

BME in the ligamentous compartment (OR 7.0, *p* < 0.005), BME depth (OR 5.9, *p* < 0.005), FMD depth (OR 4.7, *p* < 0.005), bilateral FMD (OR 4.8, *p* < 0.005) and erosions (OR 5.9, *p* < 0.005) were independently associated with axSpA (Table 4). BME in the ligamentous compartment was also a risk factor for having an iliosacral complex (OR 2.8, *p* = 0.045). BME depth (OR 3.7, *p* = 0.007) and unilateral FMD (OR 6.4, *p* < 0.005) were risk factors for having dysmorphic cartilaginous joint facets. Unilateral FMD and bilateral sclerosis were associated with female gender (OR male 0.1, *p* = 0.011 and 0.2, *p* = 0.041, respectively) and childbirths (OR 3.8, *p* = 0.023 and 4.8 *p* = 0.012, respectively). Furthermore, unilateral sclerosis (OR male 0.1, *p* = 0.011) and unilateral erosions (OR male 0.2, *p* = 0.041) were also associated with female gender. None of the MRI variables was associated with bipartite iliac bony plate, lumbar disc extrusion, increasing age or BMI.

**Table 4:** Univariate logistic and, when appropriate, linear regressions of the association between MRI patterns and axSpA diagnosis, anatomical sacroiliac joint variations, gender, childbirths, age and body mass index. Odds radios (confidence intervals) if other not stated

	axSpA	acc SIJ	ISC	Dysm CF	BIBP	Extrusion	Gender	Childbirths	Age^a^	BMI^a^
**Unilateral BME**	0.6 (0.2–1.8)	0.5 (0.2–1.3)	0.6 (0.2–1.4)	0.6 (0.2–1.7)	1.0 (0.3–3.2)	1.8 (0.7–5.1)	0.5 (0.2–1.5)	1.4 (0.6–3.6)	0.0 (− 2.7–2.7)	− 1.1 (− 3.2–0.9)
**Bilateral BME**	2.0 (0.7–5.8)	1.4 (0.5–3.4)	1.6 (0.7–4.1)	2.0 (0.8–5.4)	1.2 (0.4–3.9)	0.4 (0.2–1.1)	1.5 (0.6–3.7)	0.8 (0.3–1.9)	− 0.8 (− 3.5–1.8)	1.5 (− 0.4–3.5)
**Unilateral FMD**	0.6 (0.2–2.1)	0.3 (0.1–1.0)	0.8 (0.3–2.3)	**6.4 (2.0**–20.5)*	2.8 (0.9–9.2)	1.1 (0.3–3.6)	**0.1 (0.0**–0.6)*	**3.8 (1.2**–11.8)*	0.9 (− 2.1–4.0)	− 0.6 (− 2.9–1.7)
**Bilateral FMD**	**4.8 (1.8**–13.1)*	1.4 (0.6–3.5)	0.8 (0.3–1.9)	0.8 (0.3–2.0)	0.8 (0.3–2.7)	0.5 (0.2–1.4)	1.6 (0.7–4.1)	1.0 (0.4–2.6)	− 0.8 (− 3.4–1.8)	1.9 (− 0.1–3.9)
**Unilateral sclerosis**	0.6 (0.2–2.1)	1.4 (0.5–4.1)	1.5 (0.5–4.4)	1.4 (0.5–4.0)	1.3 (0.4–4.6)	0.1 (0.0–1.0)	**0.1 (0.0**–0.6)*	2.0 (0.7–5.8)	− 1.7 (− 4.8–1.3)	− 0.8 (− 3.1–1.6)
**Bilateral sclerosis**	1.4 (0.4–4.3)	0.7 (0.2–2.0)	2.6 (0.8–8.9)	1.6 (0.5–4.7)	3.1 (0.9–10.3)	0.5 (0.1–2.1)	**0.2 (0.1**–0.9)*	**4.8 (1.4**–16.3)*	1.2 (− 2.9–4.3)	2.2 (− 0.1–4.5)
**Unilateral erosion(s)**	2.6 (0.9–7.9)	0.3 (0.1–1.1)	1.0 (0.3–2.8)	2.2 (0.7–6.3)	1.4 (0.4–5.1)	0.5 (0.1–2.1)	**0.2 (0.1**–0.9)*	1.8 (0.6–5.1)	− 0.5 (− 3.7–2.6)	− 1.1 (− 3.5–1.3)
**Bilateral erosion(s)**	**5.9 (2.0**–17.4)*	1.5 (0.5–4.0)	1.0 (0.4–2.9)	0.6 (0.2–1.8)	0.4 (0.1–1.9)	0.2 (0.1–1.2)	**2.2 (0.8**–6.0)*	0.9 (0.3–2.4)	− 1.3 (− 4.2–1.6)	1.2 (− 1.0–3.4)
**Ligamentary BME**	**7.0 (2.5**–19.6)*	1.9 (0.8–4.8)	**2.8 (1.0**–7.6)*	0.9 (0.3–2.2)	1.3 (0.4–3.9)	0.4 (0.1–1.2)	1.4 (0.6–3.6)	1.2 (0.5–2.9)	1.3 (− 1.4–3.9)	1.4 (− 0.6–3.4)
**BME depth**	**5.9 (2.1**–16.3)*	0.7 (0.3–1.8)	1,2 (0.5–3.2)	**3.7 (1.4**–9.7)*	1.0 (0.3–3.1)	0.4 (0.1–1.3)	1.0 (0.4–2.6)	0.8 (0.4–2.1)	0.2 (− 2.5–2.9)	0.0 (− 1.1–3.1)
**FMD depth**	**4.7 (1.7**–13.1)*	1.2 (0.5–3.1)	2.0 (0.7–5.5)	1.6 (0.6–4.1)	0.8 (0.2–2.8)	0.3 (0.1–1.2)	1.4 (0.5–3.5)	0.9 (0.4–2.3)	0.1 (− 2.7–2.9)	1.0 (− 1.1–3.1)

*Acc SIJ* accessory sacroiliac joint, *axSpA* axial spondyloarthritis, *BIBP* bipartite iliac bony plate, *BME* bone marrow edema, *BMI* body mass index, *dysm CF* dysmorphic cartilaginous facets, *extrusion* lumbar disc extrusion, *FMD* fatty marrow deposition, *ISC* iliosacral complex

*p < 0.05

^a^Linear regressions and results reported as regression coefficients (confidence intervals)

## Discussion

In this study, we compared the topographical location and volume of MRI lesions in patients with axSpA and MBP fulfilling or almost fulfilling the ASAS criteria in the early diagnostic state. This is highly clinically relevant, as it is at this time point radiologists and clinicians are challenged, especially in patients with MRI changes suggestive of axSpA. This is underlined by the result of the global assessment of MRI by two experienced musculoskeletal radiologists (Table 2). In this assessment, a not insignificant part of axSpA patients had scores below 0 (deemed not to have axSpA), and some MBP patients had scores above 0 consistent with the radiologist being confident regarding the presence of axSpA based on the MRI appearance.

One-plane MRI assessment with semi-coronal T1 and STIR sequences of the cartilaginous joint compartment is currently most used in the diagnostic assessment in axSpA as well as in axSpA monitoring such as the SPARCC [20] and Berlin [29] methods, with references to the 2009 ASAS definitions [4]. Weber and colleagues demonstrated by adding the semi-axial scan plane to the assessment an overall decrease in the BME prevalence along with fewer lesions observed in the lower ilium [18], indicating that some of the observed ‘BME lesions’ could be due to partial volume effects from vessels and anatomical variations. This is important in the initial diagnostic phase to reduce false-positive BME findings, possibly incorrect diagnosis and overtreatment. As the SIJ morphology varies [30], it is possible that by using solely one-plane assessment, the location of BME in the cartilaginous compartments and the detection in the ligamentous compartments, respectively, is limited as it is difficult to gain an overview of the entire joint topography.

We demonstrated, when using the two-plane scoring system, that patients with MBP had BME predominantly in the middle anterior sacrum (Fig. 1B), followed by the upper anterior sacrum. In one previous study, Hoballah et al. [14] reported BME prevalence divided into upper, middle and lower joint portions according to an axial plane, comparable to our study method where we used the semi-axial plane for scoring supported by findings on the semi-coronal sequences. They compared nulliparous with postpartum women and demonstrated a BME prevalence of 14% in nulliparous and 21–33% in postpartum women of which 65–87% was located in the middle joint portion (sacrum and ilium collapsed) in line with our results in the MBP group. As suggested by Weber et al., this may represent a ‘strain-related’ BME pattern [7, 18].

AxSpA patients had high BME sumscores in the same locations as the MBP patients, but at the same time, they presented with a widespread pattern both anteriorly and posteriorly (Fig. 1B) in accordance with the previous findings [2, 6, 8, 12]. Another main finding in axSpA patients was the high BME scores in the ligamentous joint compartments, an infrequent finding in the MBP group, also supported by high OR of having axSpA in case of BME in the ligamentous compartment (Table 4). Another study by Weber et al. did not find any additional diagnostic value by adding observed BME in the ligamentous compartment to that of the cartilaginous compartment. However, in accordance with our findings, they found high BME prevalence ranging between 12 and 80% in non-radiological axSpA and ankylosing spondylitis but only in 2–6% of healthy controls and patients with non-specific back pain, respectively [31].

As previously reported by Molto et al. [8], our group of axSpA patients also had widespread FMD and erosions (Figs. 2A and 3C), confirmed in the regression analyses where both bilateral FMD and erosions were risk factors of an axSpA diagnosis. Furthermore, significantly more axSpA patients had the presence of BME and FMD depth in most locations, even though also present in the MBP group.

The between-group differences in the distribution of SIJ normal variations and lumbar spine findings did not reveal major differences (Table 3). This is inconsistent with a recent CT study, including four of the same variations analysed in the present study, demonstrating a significantly higher prevalence of atypical SIJ morphologies in patients with mechanical joint disease compared to axSpA patients and healthy controls [16]. With an overall prevalence of five different SIJ variations, including the lumbo-sacral transitions vertebra, of 80% and 90% in the axSpA and MBP groups, respectively, one can argue that we are overcalling them by too wide definitions, and some of the atypical morphologies may simply represent not well-described normal anatomy. On the other hand, recently, we did a CT-MRI comparison study on atypical SIJ morphologies including seven different variations in healthy young adults (mean age of 28 years) using the same definitions as in this study [19]. Here, we detected variations by MRI in 55% of the participants, with a remarkable lower prevalence of accessory SIJ (14%), iliosacral complex (16%), dysmorphic cartilaginous joint facets (14%) and bipartite iliac bony plate (12%) compared to the present results. In the present, axSpA patients’ atypical SIJ morphologies were often accompanied by BME and/or structural changes. One theory explaining this, as elaborated by Jacques et al. [32], is that variations may induce micro-trauma causing inflammation in predisposed persons such as axSpA patients.

Our study has limitations. We had a small sample size of 84 patients. Furthermore, the MDT conference approach of axSpA diagnostics has not been compared to a traditional expert-opinion; however, one of the rheumatologists attending the MDT conferences had examined 60% of the patients. Additionally, it is notable that our MBP group had high morbidity as they fulfilled or almost fulfilled the 2009 ASAS classification criteria being a subgroup of a much larger cohort of patients with low back pain for 2–12 months. This made a comparison to other studies including patients with non-specific low back pain not meaningful. Despite this, we clearly demonstrated the differences in location and volume of MRI lesions. The MRI scoring system is not validated but compared to previous studies demonstrated acceptable interreader agreements [1, 33, 34]. Further studies are needed to confirm the diagnostic value of two-plane assessment of the SIJ. As a T1 semi-axial sequence was not performed in this research project, it was not possible to perform an as detailed assessment of FMD, sclerosis and erosions as for BME.

## Conclusions

In conclusion, axSpA patients had a widespread distribution of BME with high volumes, whereas BME in the MBP group predominantly were located in the middle anterior part of the sacrum, demonstrating a ‘strain-related’ BME pattern. Furthermore, BME in the ligamentous compartment was almost exclusively seen in axSpA, which also demonstrated a widespread distribution of structural changes. In the early diagnostic period, these findings may contribute to the distinction between axSpA and MBP patients with SIJ MRI changes and thereby facilitate the axSpA diagnosis.

## Abbreviations

ASAS: Assessment of SpondyloArthritis International Society
axSpA: Axial spondyloarthritis
BME: Bone marrow edema
FMD: Fatty marrow deposition
ICC: Intraclass correlation
MBP: Mechanical back pain
OR: Odds ratios
SIJ: Sacroiliac joint
SpA: Spondyloarthritis
STIR: Short tau inversion recovery
